# Use of Anticancer Therapies and Economic Burden Near the End of Life in Japan: Results From Claims Database

**DOI:** 10.1200/GO.22.00227

**Published:** 2022-12-01

**Authors:** Yusuke Kajimoto, Kazunori Honda, Kazuki Nozawa, Mineko Mukai, Lida Teng, Ataru Igarashi

**Affiliations:** ^1^ Department of Health Economics and Outcomes Research, Graduate School of Pharmaceutical Sciences, The University of Tokyo, Tokyo, Japan; ^2^ Oncology Science Unit, MSD. K.K., Tokyo, Japan; ^3^ Department of Clinical Oncology, Aichi Cancer Center Hospital, Nagoya, Japan; ^4^ Nursing Department, Aichi Cancer Center Hospital, Nagoya, Japan; ^5^ Unit of Public Health and Preventive Medicine, Yokohama City University School of Medicine, Yokohama, Japan

## Abstract

In patients with cancer, aggressive treatment near the end of life (EOL) may decrease quality of life and increase medical costs. In this study, we examined the use of anticancer therapies near the EOL in Japan.

**METHODS:**

We used a commercial database of health insurance claims in Japan, to examine patient data on cancer and death until August 2020. We assessed the proportion of patients using anticancer therapies within 14 days of death, associated factors, and medical costs from the payer's perspective.

**RESULTS:**

The database documented 5,759 patients with cancer who died between December 2013 and August 2020. Among them, 4.8% of patients and 3.9% of age-adjusted patients received anticancer therapy within 14 days of death. Patients age < 60 years were associated with a high probability of receiving anticancer therapy near the EOL. The estimated annual anticancer therapy and related costs were Japanese yen 1,296 million (US dollars 12.6 million).

**CONCLUSION:**

We found the percentage of patients receiving anticancer therapies within 14 days of death in Japan, its associated factors, and economic burden. Our findings can serve as a benchmark for optimizing EOL care.

## INTRODUCTION

Patients with advanced cancer are administered anticancer therapy as a potential cure, as well as to prolong survival and improve quality of life (QOL).^1^ However, anticancer therapies might decrease QOL in patients with a poor Eastern Cooperative Oncology Group performance status, given reduced response rates and associated toxicity.^2-4^ The National Quality Forum and ASCO Quality Oncology Practice Initiative suggest that minimizing aggressive treatment near the end of life (EOL), especially in the last 14 days of life, may enhance QOL and reduce costs.^5,6^ Additional lines of therapy are currently used compared with the past; more patients may receive anticancer therapies near the EOL.^7,8^ Recently, many targeted therapies have been developed and their costs have been relatively high.^9,10^ For gastric cancer treatment, prices as of March 2021 are Japanese yen (JPY) 362,032 (US dollars [USD] 3,520) for ramucirumab (500 mg) and JPY 413,990 (USD 4,026) for nivolumab (240 mg) as targeted therapies, and JPY 1,095 (USD 11) for fluorouracil (1,000 mg) and JPY 7,747 (USD 76) for cisplatin (50 mg/100 mL) as nontargeted therapies.^10,11^ The use of anticancer therapies near the EOL might present an economic burden for patients/patient's families and the health care system.

**CONTEXT**

**Key Objective**
In patients with cancer, aggressive treatment near the end of life may decrease quality of life and increase medical costs. How many patients with cancer receive anticancer therapy near the end of life in Japan?
**Knowledge Generated**
Among our data, 4.8% of patients and 3.9% of age-adjusted patients received anticancer therapy within 14 days of death. The estimated annual anticancer therapy and related costs were Japanese yen 1,296 million (US dollars 12.6 million).
**Relevance**
We showed the percentage of patients receiving anticancer therapies within 14 days of death in Japan and the economic burden. Our findings can serve as a benchmark for optimizing end-of-life care.

Several reports have highlighted the proportion of patients using anticancer therapy near EOL in Canada, China, the United States, and Japan,^6,12-17^ indicating the need to optimize EOL care. Although previous Japanese studies exist, the sample size is limited. A larger cohort is required to determine a more accurate situation for EOL cancer care in Japan. Additionally, data regarding the economic burden of anticancer therapy near EOL are required, as it is an essential decision-making factor in health policy.

In this study, we aimed to determine the proportion of patients who received anticancer therapy near EOL and to establish the associated factors. Furthermore, we estimated the medical costs of anticancer therapies used within 14 days of death from the payer's perspective.

## METHODS

### Study Design and Data

This retrospective study was performed using a claims database, Medi-Scope (INTAGE Real World, Inc, Tokyo, Japan), which is a commercial database comprising public health insurance claims mainly covering employees and their dependents in Japan.^18^ We assessed all data available in Medi-Scope, with August 2020 as the cutoff. We identified patients with any cancer, including hematologic malignancies, and disease names without a suspicious disease flag and death as outcome information in the database. A suspicious flag is used before diagnosis and is not used after diagnosis. Patients whose disease codes included C00 to C97 of the International Classification of Diseases (ICD-10) codes were classified as patients with cancer in this study.^19^ Furthermore, patients with no data were excluded from the analysis. The death month was defined as the month of the death record, and the death date was defined as the date of each patient's last claims data. Age was calculated as the date of death and birth. Data on causes of death were not included in the database.

We classified anticancer therapies into two categories: targeted therapy and nontargeted therapy. Targeted therapy includes immune checkpoint inhibitors, antibody-drug conjugates, monoclonal antibody, small molecules, hormones, retinoids, histone deacetylase inhibitors, and vascular endothelial growth factor inhibitor (aflibercept beta).^20^ Nontargeted therapy includes the other anticancer therapies, such as cytotoxic therapy. We refer to all therapies, consisting of targeted and nontargeted therapies, as any anticancer therapy.

### Ethical Approval

The study protocol and all amendments were approved by the appropriate ethics committee of the University of Tokyo (31-33).

### Informed Consent

No informed consent was required for study participation, as this study was performed using a database.

### Outcomes

We determined the proportion of patients using targeted, nontargeted, or any anticancer therapies within 14, 30, 60, 90, 120, 150, or 180 days of death and examined age-adjusted data for deaths of patients with cancer in Japan in 2018.^21^ Age-adjustment used the following age categories: < 40, 40-49, 50-59, 60-69, and ≥ 70 years.

Differences between patients using anticancer therapies within 14 days of death and other patients were assessed on the basis of age at death, sex, year of death, region, number of beds per hospital, hospital organization, medicine categories, and cancer lesions. The characteristics of patients who started anticancer therapy within 14 days of death were investigated.

### Cost Analysis

We calculated the anticancer therapy costs and any related costs per patient using the available data set from the payer's perspective. The anticancer therapy–related costs consisted of companion diagnosis, administrative procedures, and management of anticancer therapies but did not include costs for managing adverse events. Additionally, we estimated the annual costs of targeted and nontargeted therapies, as well as anticancer therapy–related costs within 14 days of death. The cost estimation used age categories similar to the outcomes (< 40, 40-49, 50-59, 60-69, and ≥ 70 years) to adjust for age differences in cancer deaths in Japan in 2018.^21^ One-way sensitivity analysis was implemented to illustrate the uncertainty due to cancer medicine costs, anticancer therapy–related costs, percentage of patients receiving anticancer therapies within 14 days of death, and the annual number of cancer deaths in Japan. A 95% CI was used for each range. The annual number of cancer deaths was obtained using the Cancer Statistics for Japan between 2014 and 2019.^21^ Furthermore, other parameters were obtained from this study. The Purchasing Power Parities presented by the Organization for Economic Cooperation and Development statistics in 2020 was used as the exchange rate (USD 1 = JPY 102.84).^22^

### Statistical Analyses

We examined the factors associated with anticancer therapy within 14 days of death by using Pearson's chi-squared test for univariate analysis among all deceased patients. The factors included age at death, sex, region, year of death, number of beds per hospital, and hospital organization. Factors presenting significant differences in the univariate analysis were evaluated using multivariate analysis. Statistical comparisons were two-sided, and a *P* value < .05 was considered statistically significant. Data analyses were performed using R version 3.5.2 (The R Foundation for Statistical Computing, Vienna, Austria).

## RESULTS

### Data Set and Patient Characteristics

The database examined contained 7,423,105 patient claims from December 2013 to August 2020. Among the 215,872 patients recorded as having cancer, 5,774 died (outcome data). Fifteen patients were excluded because of incomplete data available for the analysis. Accordingly, 5,759 patients were included in the analysis. The median age at death was 57 years (range, 0-75 years). The number of patients who received anticancer therapy was 3,946 (68.5%). Patient background information is presented in Table 1.

**TABLE 1:**
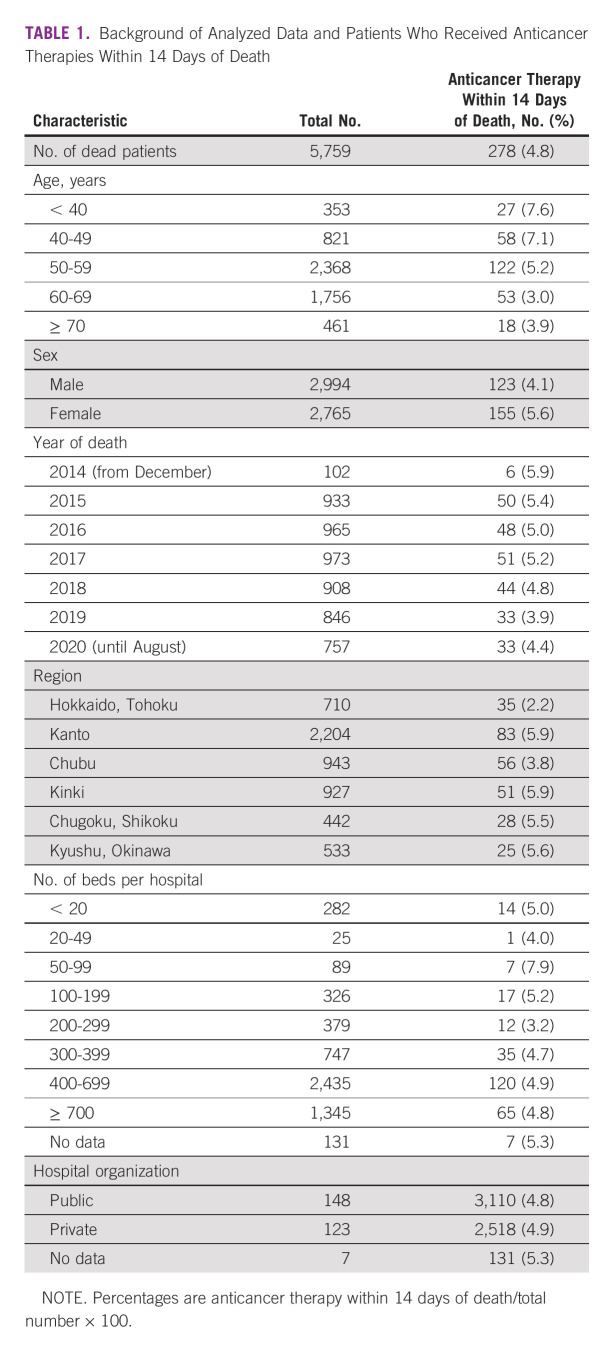
Background of Analyzed Data and Patients Who Received Anticancer Therapies Within 14 Days of Death

### Frequency of Anticancer Therapy

**FIG 1:**
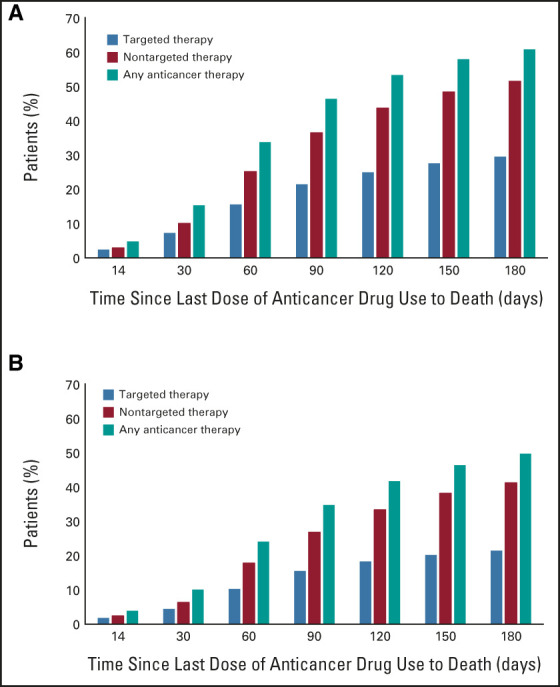
Percentage of patients using anticancer therapies within 14-180 days of death: (A) no adjustment and (B) age-adjusted for cancer-related deaths in Japan in 2018. Targeted therapies include immune checkpoint inhibitors, antibody-drug conjugates, and other targeted therapies. Nontargeted therapies include other anticancer therapies such as cytotoxic and hormone therapies. Any anticancer therapy consists of targeted and nontargeted therapies. Patients who received both targeted and nontargeted therapies were included in all the categories.

The frequencies of targeted, nontargeted, or any anticancer therapy within 14 days of death were 2.5%, 3.0%, and 4.8%, respectively (Fig 1). Age-adjusted frequencies were 1.8%, 2.4%, and 3.9%, respectively. Furthermore, the frequencies of any anticancer therapy with no adjustment were 15.3%, 33.8%, 46.4%, 53.4%, 58.0%, and 60.9% within 30, 60, 90, 120, 150, and 180 days, respectively, and their age-adjusted frequencies were 10.1%, 24.1%, 34.9%, 41.7%, 46.4%, and 49.8%, respectively.

The thyroid and other endocrine glands (11.3%), breast (9.1%), and lymphoid, hematopoietic, and related tissues (8.6%) revealed a high percentage of anticancer therapy within 14 days of death (Table 2).

**TABLE 2:**
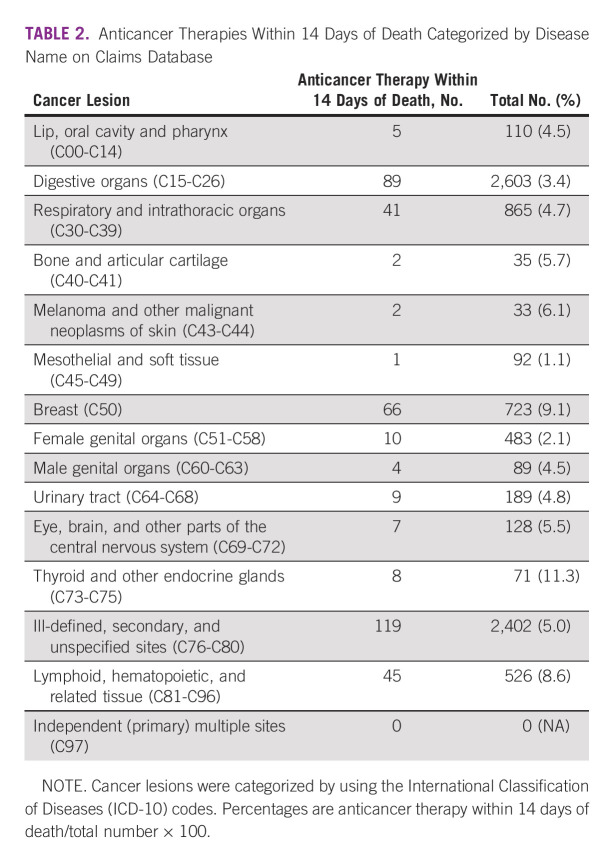
Anticancer Therapies Within 14 Days of Death Categorized by Disease Name on Claims Database

Table 3 presents the percentage of patients according to the category of medicine used within 14 days of death. Other nontargeted therapies, such as arsenic trioxide, lenalidomide hydrate, OK‐432, pomalidomide, talaporfin sodium, thalidomide, trabectedin, and ubenimex, showed the highest percentage (8.7%; 6/69) among nontargeted therapies. Among targeted therapies, other targeted therapy showed the highest percentage (11.4%; 4/35), followed by immune checkpoint inhibitors (6.4%; 19/295), small molecule (6.4%; 46/719), hormone therapy (5.9%; 26/440), and antibody‐drug conjugates (4.6%; 3/65).

**TABLE 3:**
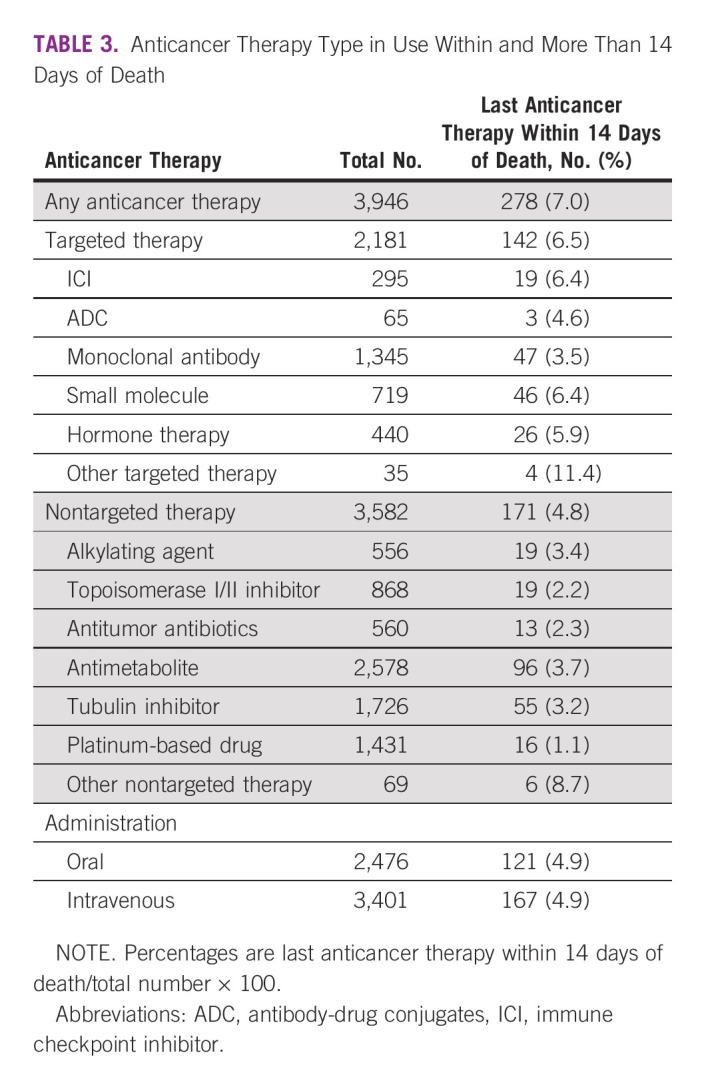
Anticancer Therapy Type in Use Within and More Than 14 Days of Death

### Factors Associated With Anticancer Therapy Within 14 Days of Death

Patients age < 60 years at death (*P* < .001) or male (*P* = .010) were more likely to receive anticancer therapies within 14 days of death following univariate analysis (Table 4). In the multivariate analysis, patients age < 60 years (*P* < .001) were more likely to receive any anticancer therapy within 14 days of death.

**TABLE 4:**
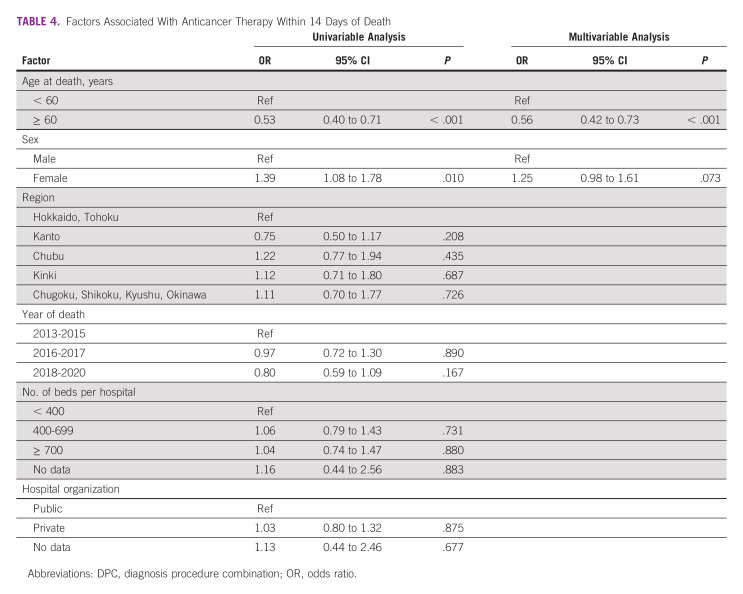
Factors Associated With Anticancer Therapy Within 14 Days of Death

### Characteristics of Patients Who Started Anticancer Therapy Within 14 Days of Death

Twenty-six patients started anticancer therapy within 14 days of death. The median age of the patients was 59 years (range, 23-74 years). The numbers of male and female were 18 and 8 patients, respectively. Among them, 10 patients had hematologic malignancies and 16 patients had solid tumors. Regarding hematologic malignancy, four patients received combination therapy including cytarabine, and three patients received other combination therapies including rituximab, ponatinib hydrochloride, or brentuximab vedotin. The other three patients received monotherapy. For solid tumors, four patients received combination therapy, such as platinum-doublet or ifosfamide plus etoposide, and the other 12 patients received monotherapy.

### Cost Analysis

**FIG 2:**
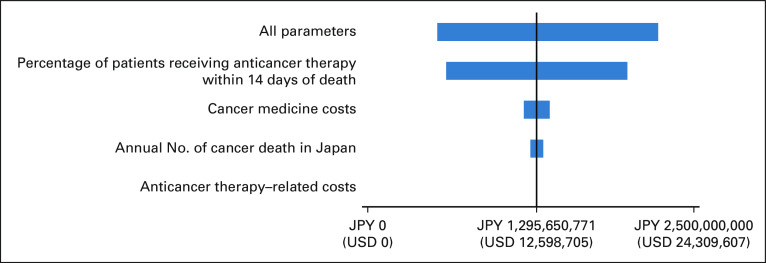
Sensitivity analysis of annual economic burden following anticancer therapy within 14 days of death. The parameter ranges were 95% CIs of anticancer therapy costs, anticancer therapy–related costs from all data in this study, the percentage of patients using anticancer therapies within 14 days of death from 2014 to 2019 in this study data set, and the annual number of cancer deaths in Japan from 2014 to 2019 from Foundation for Promotion of Cancer Research.^21^ JPY, Japanese yen; USD, US dollars.

The costs within 14 days of death per patient were JPY 81,499 (USD 792), which included JPY 78,207 (USD 760) for cancer medicine and JPY 3,292 (USD 32) for anticancer therapy–related costs. The estimated annual economic burden in Japan was JPY 1,296 million (USD 12.6 million), which included JPY 1,128 million (USD 11.0 million) for targeted therapy, JPY 130 million (USD 1.3 million) for nontargeted therapy, and JPY 38 million (USD 0.4 million) for anticancer therapy–related costs. The most influential factor in the sensitivity analysis was the percentage of patients receiving anticancer therapies within 14 days of death, followed by cancer medicine costs, annual number of cancer deaths in Japan, and anticancer therapy–related costs (Fig 2). Accordingly, considering all parameters, the maximum accrued cost was JPY 2,226 million (USD 21.7 million) and the minimum cost was JPY 532 million (USD 5.2 million).

## DISCUSSION

In this study, we observed that 4.8% of patients with no adjustment and 3.9% of patients with age-adjustment received anticancer therapies within 14 days of death. Minimizing low-value care that decreases the QOL of patients with cancer near EOL remains an important challenge. Our results concerning the percentage of patients receiving anticancer therapies within 14 days of death were slightly higher than those in other Japanese studies (3.1% and 3.3%, respectively).^16,17^ In those studies, eight patients from a single site and 41 patients from the database data were analyzed. These small numbers could explain the discrepancies. For countries other than Japan, the percentages were as follows: 5.8%-15.7% in the United States,^6,14,15^ 2.02%-2.88% in Canada, and 9% and 5.3% in China.^13^ Various study populations in terms of cancer lesion, country/culture, database, or hospital could underlie these substantial differences. Using these results as a benchmark, future studies should attempt to confirm the optimal percentage within each setting on the basis of all results, including ours.

Platinum-based therapies are frequently used as first-line therapy.^8,11^ On the basis of these results, platinum-based therapies are less frequently used than other therapies within 14 days of death. Moreover, a higher frequency was seen in hormone therapy, which is often used for luminal breast cancer.^23^ It is considered to contribute to the higher frequency in breast cancer (9.1%). Further studies are needed to assess whether targeted therapies and hormone therapy can benefit patients during EOL care. We observed that anticancer therapies were more likely to be administered near EOL in patients with hematologic malignancies (8.6%). This finding was consistent with those of previous studies in the United States, Canada, and Japan.^12,15,16^ Patients who started anticancer therapy within 14 days of death received strong combination therapies, such as cytarabine, in our study. These therapies are expected to achieve immediate complete remission for hematologic malignancy even in patients with severe conditions.^24^ However, treatment-related death can occur among the patient popuation.^25^ The use of anticancer therapies in hematologic malignancies should be justified on the basis of risk and benefit considerations, although this application occurs near EOL.

Age was associated with receiving anticancer therapies within 14 days of death. This result is consistent with that of most other research studies.^6,12-15,17^ Although younger patients with cancer are likely to be in a better clinical condition than older ones in palliative care settings,^26^ clinical condition should be carefully assessed to avoid QOL deterioration or treatment-related toxic death. Palliative care or shared decision making can help reduce aggressive care and improve outcomes.^27,28^

In this study, we determined that the estimated annual economic burden of anticancer therapies and related costs was JPY 1,296 million (USD 12.6 million). In 2018, the annual medical care expenditure in Japan was JPY 43.7 trillion (USD 424.9 billion), while the expenditure for cancer alone was JPY 4.5 trillion (USD 43.8 billion).^29^ Following sensitivity analysis, the maximum economic burden conferred by cancer expenditures was only 0.05%. Accordingly, the economic burden of anticancer therapy within 14 days of death minimally affected the health care system in Japan. However, out-of-pocket costs can considerably burden patients and their families. Unnecessary expenditures should be reduced.

Our study has considerable strengths, including a large sample size from all over Japan and details of the claims data, including cost information. However, a few limitations should be noted, including the characteristics of the claims data and the database. As shown in the patient characteristics, the age distribution did not represent the general population in Japan, and the cohort population had only a few elderly individuals, as the database was mostly composed of employees and their dependents. To address this limitation, we conducted an age-adjusted analysis. The claims data do not list treatments not covered by public insurance and hence fail to include uninsured treatments, such as clinical trials. Additionally, claims data do not include clinical data, such as reasons for death, laboratory test results, and patient status. As shown in Table 2, some of the patients had multiple lesions. Clinical data could complement the uncertainty, bias, and interpretation of the results.

In conclusion, 3.9% of patients with cancer received anticancer therapy within 14 days of death in Japan. These findings will serve as a benchmark for further research to optimize EOL care. Although it has minimal impact on national health care costs, unnecessary anticancer therapy near EOL can be a burden for patients and their families and should be avoided, especially for younger patients.

## Data Availability

The data used in this study are not available for sharing. INTAGE Real World has a right of the database.
